# Antifungal activity of paeonol against *Botrytis cinerea* by disrupting the cell membrane and the application on cherry tomato preservation

**DOI:** 10.3389/fmicb.2024.1509124

**Published:** 2024-12-02

**Authors:** Yang Jiao, Yu Zheng, Shiqin Wu, Li Zhou, Hua Jiang, Yuanhong Li, Fuxing Lin

**Affiliations:** School of Public Health, Xuzhou Medical University, Xuzhou, China

**Keywords:** paeonol, *Botrytis cinerea*, ergosterol, cherry tomato, quality

## Abstract

*Botrytis cinerea* may cause gray mold in fruits and vegetables. Paeonol, an active component of traditional Chinese medicine, could suppress various microbial growth. However, reports on its effect on *B. cinerea* have not yet been documented. In this paper, we demonstrated that paeonol completely inhibited *B. cinerea* growth at 250 mg/L, corroborated by the observation of irregular morphological alterations in *B. cinerea* exposed to paeonol. Notably, the investigation of the operating mechanism revealed that paeonol induced cell death by disrupting the cell membrane, potentially mediated by the interaction between paeonol and ergosterol from the membrane. Further studies indicated that paeonol decreased ergosterol content and the expression of certain genes involved in ergosterol biosynthesis was significantly downregulated. In addition, paeonol treatment reduced the gray mold of cherry tomatoes. Meanwhile, compared to the control treatment, paeonol treatment could reduce weight loss and maintain higher contents of total soluble solid (TSS) and ascorbic acid, leading to a higher quality of the stored cherry tomato. Together, the data indicate that paeonol was effective as an alternative agent targeting disrupting the cell membrane to control gray mold and prolong the shelf life of cherry tomatoes, suggesting that paeonol could be used as a natural antifungal compound during postharvest storage.

## 1 Introduction

*Botrytis cinerea*, a typical broad host necrotrophic pathogen, causes gray mold rot in more than 200 economically important crops including strawberry, tomato, grape, blueberry, and raspberry (Sarven et al., 2020; Orozco-Mosqueda et al., 2023). It can infect leaves, stems, and fruits of crops, either through direct penetration or through wounds, seriously affecting yield and quality (Rodríguez and Rodríguez, 2014). It is estimated that *B. cinerea* causes losses of $10 billion to $100 billion annually worldwide (Weiberg et al., 2013). Synthetic fungicides effectively control *B. cinerea* (Orozco-Mosqueda et al., 2023). However, excessive application of synthetic fungicides is harmful to human health and the environment, and may lead to the emergence of fungicide-resistant strains (Shao et al., 2021). Therefore, it is urgent to develop new kinds of fungicides that are safe, efficient, biocompatible, and environmentally friendly to control *B. cinerea*.

Promising results have been obtained using plant-derived natural bioactive compounds (e.g., flavonoids, alkaloids, and phenols) to control *B. cinerea* (Matrose et al., 2021). For instance, carvacrol, derived from aromatic plants, exhibits antifungal activity against *B. cinerea* with a minimum inhibitory concentration of 120 μL/L, and the anti-fungal activity can be derived from cell membrane integrity damage and cell components leakage (Zhang et al., 2019). Wang et al. (2023) demonstrated that flavonoids from *Sedum aizoon* L. (FSAL) induced a decrease in the proline, GSH, and membrane phospholipid content and reduced the expression of major genes in membrane lipid metabolism, resulting in damage the integrity and stability of the cell membrane, and finally leading to the *B. cinerea* cell death. In addition, Ma et al. (2020) found that anti-*B. cinerea* mechanism of plant-derived honokiol compound may be associated with autophagic, reactive oxygen species accumulation and mitochondrial dysfunction. Many plant-derived natural bioactive compounds have no negative impact on the environment or human health, and can be used to develop safe and sustainable antifungal products (Makhuvele et al., 2020; Khursheed et al., 2022).

Cherry tomato is a popular fruit worldwide for its nutritional value due to its high vitamins A, vitamins C, lycopene, and *β*-carotenoids (Ji et al., 2020). However, cherry tomato is susceptible to mechanical injury due to its thin exocarp and rich pulp, which increases the risk of pathogen infection (Guo et al., 2023). Gray mold, caused by *B. cinerea*, has been reported as an important postharvest disease of cherry tomato (Wu et al., 2024). Chemical fungicides and physical treatments (hot air, blue light, or temperature regulation) can effectively control gray mold on tomato during storage (Yan et al., 2022; Wei et al., 2017; Sun et al., 2024; Li et al., 2023). However, environmental pollution and fruit safety issues of chemical fungicides, high energy inputs, and costs of physical treatments restricted their use.

Paeonol, derived from *Paeonia suffruticosa* Andr and *Paeonia lactiflora* Pall, exhibits antimicrobial properties against *Klebsiella pneumonia*, *Enterobacter cloacae*, *Staphylococcus aureus*, *Listeria monocytogenes*, *Candida albicans*, and *Aspergillus flavus* (Qian et al., 2021; Zeng et al., 2022; Qian et al., 2022; Li Q. et al., 2021). Moreover, paeonol has been considered to have no obvious toxicity and can be added to pharmaceuticals, cosmetics, and health care foods (Li P. et al., 2021; Li Q. et al., 2021). Thus, paeonol provides an ideal alternative for *B. cinerea* control without food safety concerns. However, there is little information on the inhibitory effect of paeonol on *B. cinerea*, and its mechanism of action remains unclear.

In this study, we used *in vitro* and *in vivo* experiments to assess the efficacy of paeonol in inhibiting the growth and pathogenicity of *B. cinerea* on harvested cherry tomato fruit. Furthermore, using scanning electron microscopy (SEM), flow cytometry, and RT-PCR assays, we explored the potential mechanisms of action. Meanwhile, the quality of fruit treated with paeonol during postharvest storage was analyzed. These data will help determine the antifungal mechanism of paeonol and guide its application in the postharvest storage of cherry tomato.

## 2 Materials and methods

### 2.1 Strain, compound, and fruit

*Botrytis cinerea* (ACCC 37346) was purchased from the Agricultural Culture Collection of China (ACCC). Paeonol was purchased from Aladdin Industrial Corporation (Shanghai, China). Cherry tomato fruit (*Solanum lycopersicum* ‘Qian xi’) of similar maturity, size, and color and without mechanical damage or disease were purchased from commercial orchards located in Xuzhou, Jiangsu Province, China.

### 2.2 Determination of minimal inhibitory concentration (MIC)

The MIC of paeonol against *B. cinerea* was determined by sterile 96-well plate assays according to Lin et al. (2021). The absorbance was recorded at 560 nm by UV spectrophotometer (Versa max, Molecular Devices, China). MIC was set as the lowest concentration with no growth in plate assays (Fan et al., 2023). All analyses were performed with three replicates.

### 2.3 Measurement of mycelial growth and spores germination

The mycelial growth method was based on the previous study of Lin et al. (2019). *B. cinerea* mycelial disks were positioned at the center of each potato dextrose agar (PDA) plate incorporating varying concentrations of paeonol and were concurrently incubated at 23°C. The diameters of the colonies were measured at 72 h after treatment. The *B. cinerea* mycelial growth inhibition was quantified using this formula: Inhibition (%) = [control mycelial diameter (mm) − paeonol treated mycelial diameter (mm)] ÷ [control mycelial diameter (mm)] × 100%. The value of EC_50_ (concentration to inhibit 50% of mycelial growth) was calculated by linear regression of mycelial growth inhibition versus log_10_ transformation of paeonol concentrations. All analyses were performed with three replicates.

Paeonol was diluted to different concentrations in potato dextrose broth (PDB). The *B. cinerea* spore suspension was mixed with paeonol-containing PDB and incubated at 23°C for 12 h. Spore germination was determined using a microscope (BX41, Olympus, Japan). The spore germination rate was calculated based on observations of 100 spores. Triple replications were used in all analyses.

### 2.4 Scanning electron microscopy analysis

*Botrytis cinerea* mycelia exposed to paeonol at the final concentration of 0, 125, or 250 mg/L at 23°C for 12 h. Treated mycelia were re-suspended in 2.5% glutaraldehyde solution for SEM observation (Teneo *VS*, FEI Company, United States) (Lin et al., 2022).

### 2.5 Determination of paeonol mode of action

#### 2.5.1 Sorbitol assay

Sorbitol serves as an osmoregulatory agent for fungal cell wall protection (Frost et al., 1995). Cells shielded by sorbitol can proliferate in the presence of fungal cell wall inhibitors, whereas growth would be suppressed in the absence of sorbitol (Leite et al., 2014). To assess whether paeonol interacts with *B. cinerea* cell wall, the MIC against *B. cinerea* was determined using 96-well plates in medium with and without 0.8 mol L^−1^ exogenous sorbitol (Leite et al., 2014). Triple replications were used in all analyses.

#### 2.5.2 Ergosterol assay

Ergosterol, responsible for stabilizing the membrane structure via incorporation of phospholipids, represents the principal sterol constituent present in the plasma membrane of fungi, holding indispensable status for the majority of fungi (Wu et al., 2020). To determine if paeonol binds fungal membrane sterols, its MIC against *B. cinerea* in 96-well plates, with and without 400 mg/L exogenous ergosterol (Leite et al., 2014). Amphotericin B served as the positive control. Triple replications were used in all analyses.

### 2.6 Determination of the integrity of cell membrane

*Botrytis cinerea* spore suspension was exposed to 0, 125, or 250 mg/L paeonol at 23°C for 6 h. After centrifugation, spore cells were stained with propidium iodide (PI) for 15 min. Ten thousand cells were calculated and analyzed using a flow cytometry (FACS Verse, Becton, Dickinson and Company, United States).

### 2.7 Determination of calcein leakage from large unilamellar vesicles (LUVs)

To mimic the *B. cinerea* cell membrane, LUVs were synthesized by mixing phosphatidylcholine (PC)/phosphatidylethanolamine (PE)/phosphatidylinositol (PI)/ergosterol (5:4:1:2) (Cho et al., 2013). The four components were dissolved in chloroform and dried by nitrogen. LUVs-embedded calcein was created by stirring the dried LUVs in dye buffer (70 mM calcein, 10 mM Tris, 150 mM NaCl, and 0.1 mM EDTA). This solution underwent 11 cycles of freezing in liquid nitrogen and passing through polycarbonate filters with a Liposome extruder (Hand extruder, Genizer, United States). Different concentrations of paeonol were applied to calcein-encapsulating LUVs, and calcein release was measured by spectrofluorophotometer (SparkControl, Tecan, Switzerland) at wavelengths (λex = 490 nm, λem = 520 nm). Calcein leakage was calculated as: calcein leakage (%) = [(*A*_added paeonol_ − *A*_without paeonol_)/(*A*_added_ Triton X-100 − *A*_without paeonol_)] × 100. All analyses utilized three replications.

### 2.8 Determination of cellular content leakage

*Botrytis cinerea* mycelia (500 mg) were suspended in PBS and exposed to paeonol at final concentrations of 0, 125, or 250 mg/L at 23°C for 0, 2, 4, 6, or 8 h. The detection of protein, nucleic acid, and ion leakage was conducted utilizing the BCA Protein Assay Kit (Solarbio, Beijing, China), UV spectrophotometer (Versa max, Molecular Devices, China), and ICP-Optical Emission Spectroscopy (Avio 200, PerkinElmer, Waltham, United States), respectively. Each treatment was conducted in triplicate.

### 2.9 Measurement of ergosterol content

The ergosterol content of *B. cinerea* was measured as reported by Tian et al. (2012). *B. cinerea* spore suspension was inoculated into PDB containing 0, 62.5, 125, and 250 mg/L of paeonol for 4 days at 23°C. After incubation, mycelia were collected, re-suspended in 3 mL alcoholic KOH (25% w/v), and incubated at 85°C for 4 h. Sterols extraction involved adding 1 mL distilled water and 3 mL n-heptane to each sample. Then, a vigorous vortexing was conducted for 3 min to isolate the heptane layer. Analysis was performed on this layer using a UV spectrophotometer (Versa max, Molecular Devices, China) at 282 and 230 nm. Ergosterol content was calculated as: ergosterol (%) = [(*A*_282_/290)/mycelia weight] − [(*A*_230_/518)/mycelia weight]. All analyses were made with three biological replicates.

### 2.10 Ergosterol synthesis genes expression analysis

*Botrytis cinerea* spores were incubated in PDB (containing 0, 125, or 250 mg/L paeonol) at 23°C and 180 rpm for 4 days, then mycelia were lyophilized and pulverized. Total RNA was extracted with a kit (Sangon Biotech, Shanghai, China) and reverse transcribed with HiScript III All-in-one RT SuperMix Perfect (Vazyme, Nanjing, China). RT-PCR was run with Taq Pro Universal SYBR qPCR Master Mix (Vazyme, Nanjing, China) on a Step One System (7,500, Applied Biosystems, United States). The 2^−△△C(T)^ method was used for relative quantification (Livak and Schmittgen, 2001). The *Actin* gene acted as the internal control for analyzing four ergosterol synthase genes: *Erg1*, *Erg3*, *Erg10*, and *Erg13*. PCR primer pairs are shown in Table 1. Triple replications were used in all RT-PCR analyses.

**Table 1:** Sequences of primers.

Gene name	Genbank number		Primer sequence (5′ → 3′)	Size
*Actin*	XM_024697950.1	Forward	GAAGATCTTGGCTGAGCGTG	132
		Reverse	GAGGATTGACTGGCGGTTTG	
*Erg1*	XM_001547426.2	Forward	ATACCGTCGTCCTCCCAATC	178
		Reverse	CCGCCAGTTAAAGGATGACG	
*Erg3*	XM_001549088.2	Forward	GAGGCGGAAGGAGAAGATGA	136
		Reverse	TGTTCCCATTCCTCTCCCAC	
*Erg10*	XM_001550593.2	Forward	TTGTTCTCGTCTCGGAAGCT	105
		Reverse	CGGTGGTGAATTTGCTTGGA	
*Erg13*	XM_001552372.2	Forward	ACCATTGGACGTCTTGAGGT	166
		Reverse	CCCAGTTGACAGCGTTGAAA	

### 2.11 Postharvest treatment and inoculation

Cherry tomato were wounded with a sterile borer (2 mm diameter and 2 mm depth) in each fruit’s equator and then inoculated with 5 μL of *B. cinerea* spore suspension (2 × 10^4^ spores/mL). About 30 min later, each wound received 5 μL of paeonol solution at 0, 250, 500, 1,000, and 2000 mg/L. Treated fruit were then stored at room temperature and 90% relative humidity. Disease incidence was determined at 72 h after treatment. Each treatment was triplicated with 20 fruits per replicate.

### 2.12 Measurement of fruit physical and chemical properties

Cherry tomato were immersed in 0 or 2000 mg/L paeonol for 20 min, then stored under 25°C/90% humidity for 15 days. Twenty fruits were sampled at 0, 3, 6, 9, 12, and 15 days to assess physiological and quality indices.

Weight loss was measured based on previously described protocols (Lin et al., 2021). Cherry tomato were weighed at 0, 3, 6, 9, 12, and 15 days. The weight loss percentage was calculated as the difference between the initial weight and the weight recorded on the harvest day.

Total soluble solids (TSS), titratable acidity (TA), and ascorbic acid content were determined according to the method of Lin et al. (2021). The TSS result was reported as a percentage. TA was expressed as the percentage of citric acid. The ascorbic acid content was expressed as mg/Kg. All analyses were performed with three replicates.

### 2.13 Statistical analysis

Data were analyzed via ANOVA and Duncan’s multiple range with SPSS 19.0. *p* < 0.05 indicated statistical significance.

## 3 Results

### 3.1 Effect of paeonol on *B. cinerea* growth

**Figure 1:**
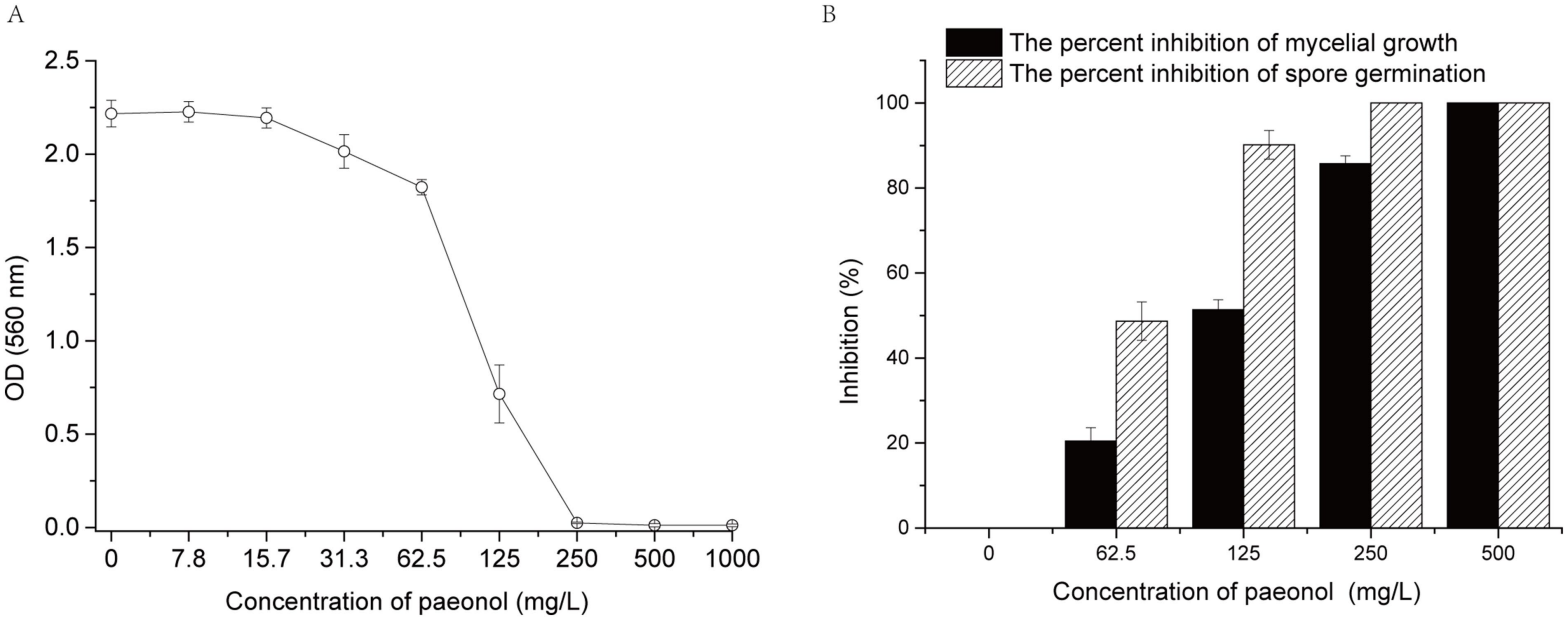
Effect of paeonol on the growth of *Botrytis cinerea in vitro*. **(A)** MIC of paeonol against *B. cinerea*. **(B)** Paeonol inhibited mycelial growth and spore germination of *B. cinerea*. Data were represented by means ± standard deviations. Experiments were carried out with three replicates.

The MIC of paeonol against *B. cinerea* was determined as a means of investigating the effect of paeonol on *B. cinerea* growth. When paeonol concentration was <125 mg/L, *B. cinerea* growth was almost unaffected. Furthermore, when paeonol concentration was ≥250 mg/L, *B. cinerea* growth was completely inhibited (Figure 1A). Thus, the MIC of paeonol against *B. cinerea* was 250 mg/L.

As demonstrated in Figure 1B, paeonol suppresses the mycelial expansion of *B. cinerea* in dose-responsive manners. The value of EC_50_ (concentration to inhibit 50% of the mycelial growth) was 120 mg/L. Mycelial growth exhibits complete suppression when paeonol concentration is elevated to 500 mg/L. Concurrently, spore germination is suppressed demonstrating analogous patterns in the presence of paeonol. These findings validate that paeonol can also effectively inhibit mycelial growth and spore germination of *B. cinerea in vitro*.

### 3.2 Effect of paeonol on the morphology and structure of *B. cinerea*

**Figure 2:**
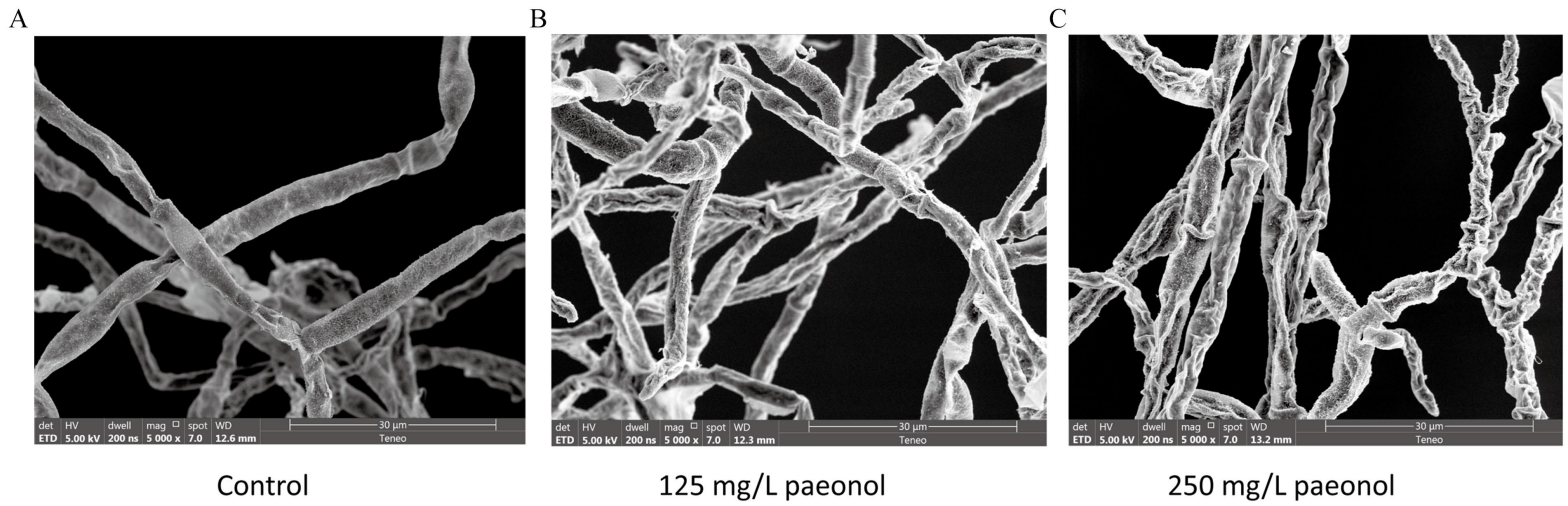
SEM images of *B. cinerea* mycelia exposed to varied concentrations of paeonol for 12 h. **(A)** Control; **(B)** 125 mg/L paeonol; and **(C)** 250 mg/L paeonol.

The SEM images showed the impact of paeonol on the morphology and structure of *B. cinerea* (Figure 2). Control group mycelia were smooth, regular, and plump, but 125 mg/L paeonol induced slight irregularities and collapse, while 250 mg/L paeonol led to severe irregularities and collapse.

### 3.3 Modes of action

#### 3.3.1 Sorbitol assay

As shown in Table 2, the MIC values of paeonol in both sorbitol and sorbitol-free media were identical, suggesting that the cell wall may not be the target of paeonol.

**Table 2:** Minimal inhibitory concentration (MIC) (mg/L) of antifungal agents in the absence and presence of sorbitol (0.8 mol/L) and ergosterol (400 mg/L) against *B. cinerea*.

Antifungal agents	Sorbitol		Ergosterol	
	Absence	Presence	Absence	Presence
Paeonol	250	250	250	500
Amphotericin B*	–	–	8	128

* Positive control. –, not tested.

#### 3.3.2 Ergosterol assay

The inhibitory effect of paeonol against *B. cinerea* was profoundly influenced by extracellular ergosterol, whereby the MIC of paeonol in the presence of ergosterol was elevated 2-fold in comparison to the absence of exogenous ergosterol (Table 2). Amphotericin B interacts with ergosterol, showing a 16-fold increase in its MIC. This result suggested that ergosterol may be the target of paeonol.

### 3.4 Effect of paeonol on membrane integrity

**Figure 3:**
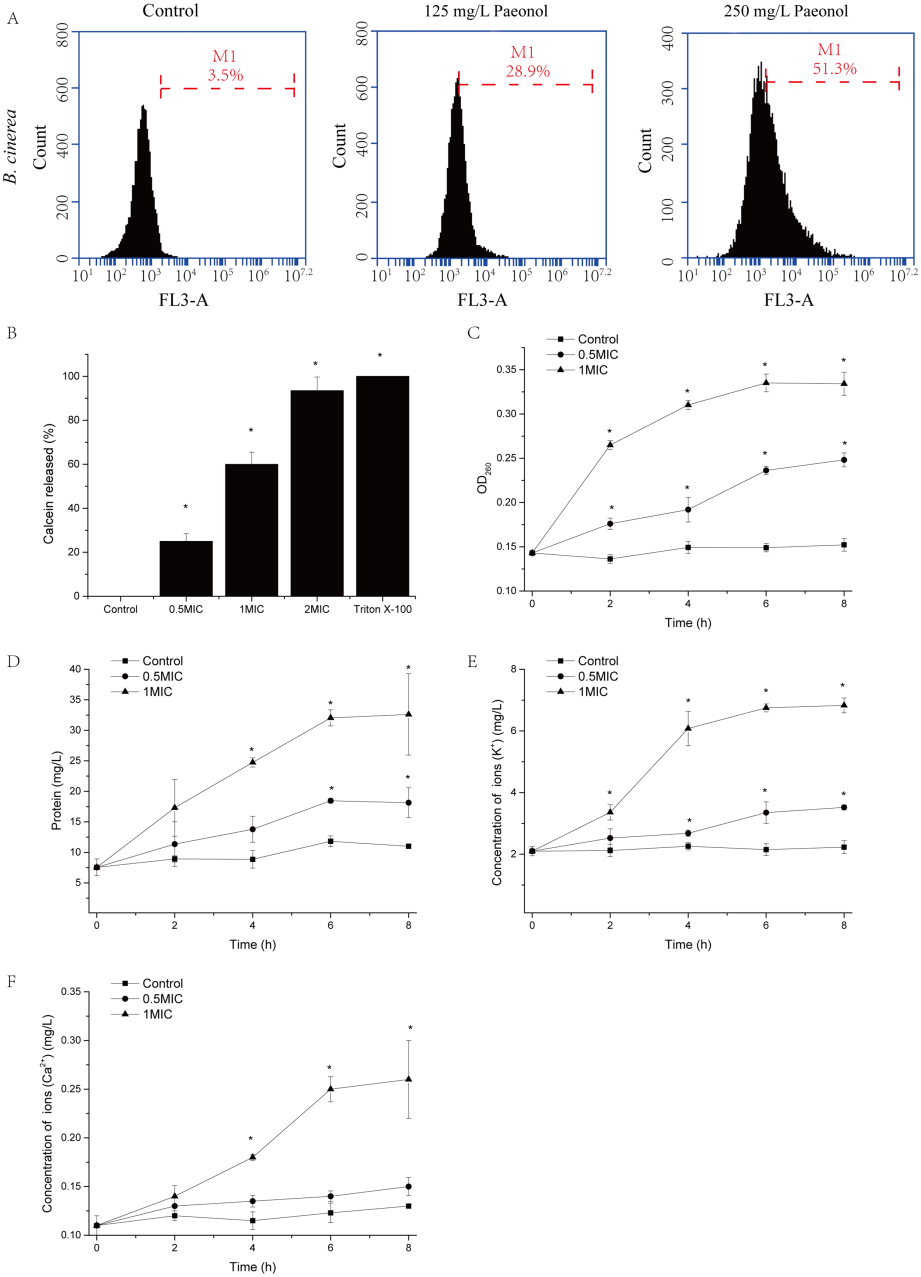
The impact of paeonol on the plasma membrane integrity of *B. cinerea*. **(A)** The PI-stained cell population of *B. cinerea* following exposure to 0, 125, and 250 mg/L paeonol for 6 h. M1, events of dead cells; 10,000 cells were calculated. **(B)** The effect of paeonol (125, 250, and 500 mg/L) on the leakage of calcein from large unilamellar vesicles. Triton X-100 solution was the positive control. The effect of paeonol (125 or 250 mg/L) on the leakage of nucleic acids **(C)**, proteins **(D)**, K^+^ **(E)**, and Ca^2+^ **(F)** over 8 h. Experiments were repeated thrice. “*” represents a significant difference (*p* < 0.05) compare to control.

Propidium iodide (PI) was used to measure cell membrane integrity as it can only enter cells that have damaged plasma membranes and are dying or already dead. The results of staining *B. cinerea* cells with PI are shown in Figure 3A. Paeonol treatment at different concentrations causes varying degrees of damage to *B. cinerea*, escalating with increasing paeonol concentration. In comparison with the control, cell damage increased from 28.9 to 51.3% when *B. cinerea* cells were exposed to 125 and 250 mg/L paeonol, respectively.

In order to confirm the ability of paeonol to disrupt fungal plasma membranes, the percentage of calcein leakage from LUVs was determined (Figure 3B). LUVs-embedded calcein was promptly liberated in a paeonol concentration-dependent manner. A concentration of 500 mg/L paeonol could instigate a leakage rate of 93.5%.

To further confirm the integrity of the cell membrane was disrupted, leakage of cytoplasm, including nucleic acids, proteins, potassium ions, and calcium ions, was measured. As shown in Figures 3C–F, nucleic acids, protein, K^+^ and Ca^2+^ of the supernatant increased in a time- and dose-dependent manner when *B. cinerea* mycelial was exposed to paeonol.

### 3.5 Effect of paeonol on ergosterol content and gene expression related to ergosterol synthesis

**Figure 4:**
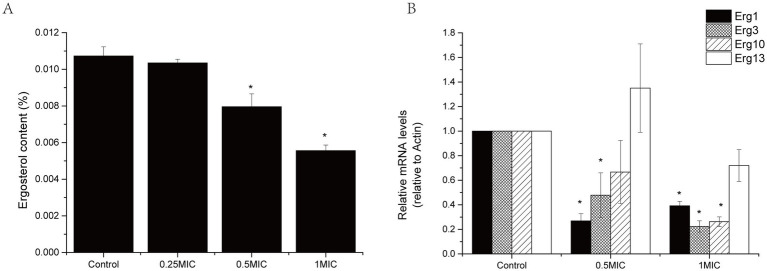
Effect of paeonol on ergosterol content **(A)** and gene expression **(B)** of *B. cinerea*. “*” indicates significant differences (*p* < 0.05) compared to control. Experiments were performed with three replicates.

The effects of paeonol on ergosterol content in the plasma membrane of *B. cinerea* are illustrated in Figure 4A. The ergosterol content of *B. cinerea* was remarkably suppressed by varying concentrations of paeonol. Following incubation of *B. cinerea* in 0.5 MIC or 1 MIC paeonol, ergosterol content was reduced by 24.7 and 48.2%, respectively, compared to the control.

Ergosterol biosynthesis is tightly regulated by Erg genes (Song et al., 2022). To gain insight into paeonol’s role in regulating ergosterol synthesis, expression levels of *Erg1*, Erg3, *Erg10*, and *Erg13* were analyzed (Figure 4B). Paeonol significantly suppressed the transcript levels of *Erg1*, *Erg3*, and *Erg10* genes.

### 3.6 Effects of paeonol on the growth of *B. cinerea* in cherry tomato

**Figure 5:**
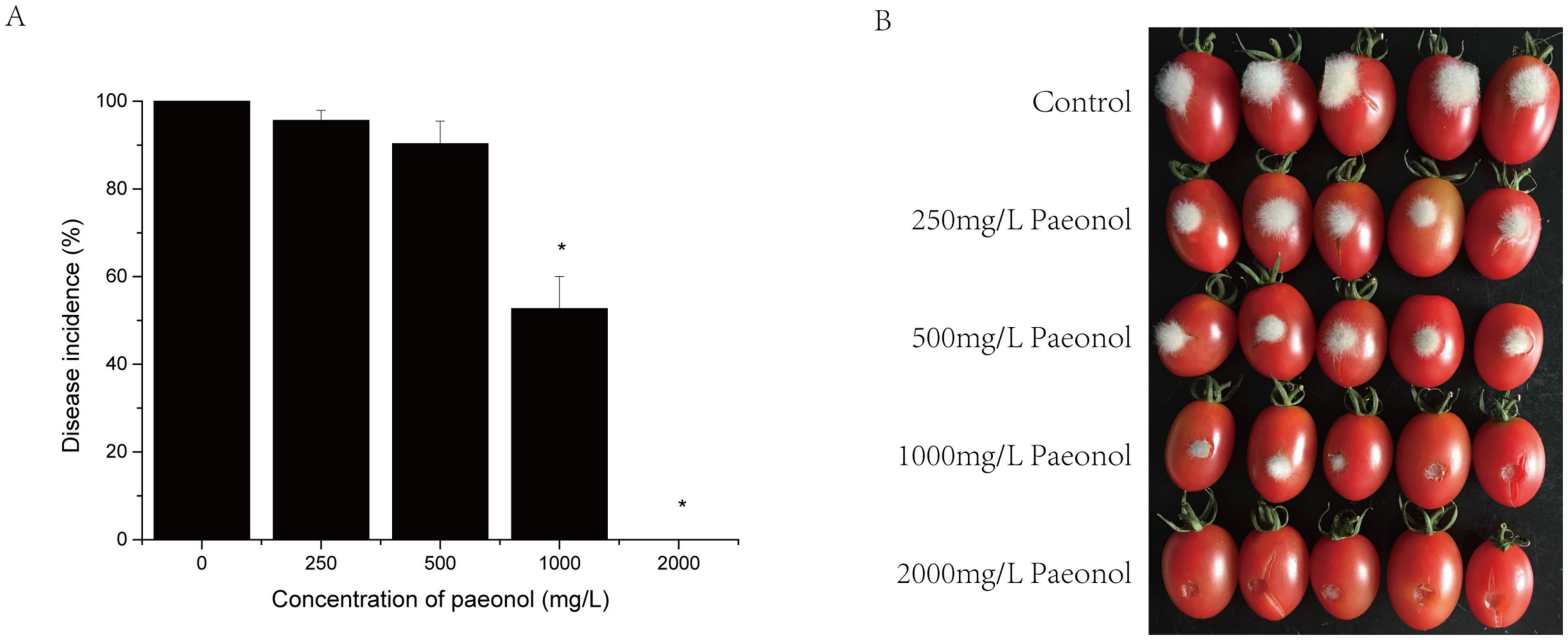
Effect of different concentrations of paeonol on the growth of *B. cinerea* in cherry tomato fruit. **(A)** Disease incidences; **(B)** representative photographs.

Considering that paeonol showed effective antifungal effects against *B. cinerea in vitro*, cherry tomato fruit was treated with paeonol during the infection assay. The incidence of gray mold decreased slightly when paeonol concentration increased from 0 to 500 mg/L at 72 h after the inoculation of the pathogen (Figure 5), with no significant difference between them (*p* > 0.05). However, disease incidence decreased significantly when paeonol concentration was increased to 2,000 mg/L (*p* < 0.05) as gray mold was completely inhibited.

### 3.7 Effects of paeonol on cherry tomato fruit quality

**Figure 6:**
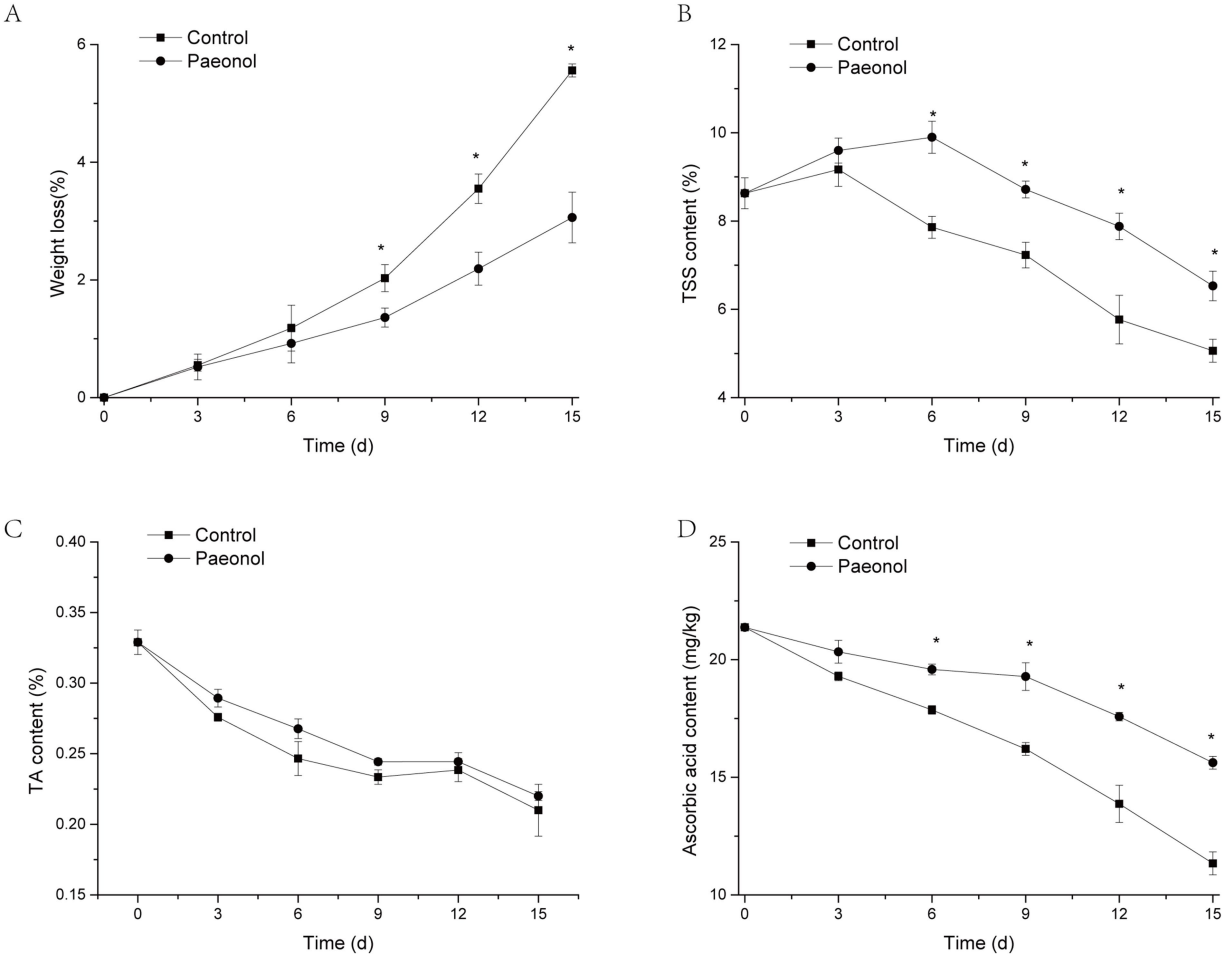
Paeonol improves the quality of cherry tomato. Weight loss **(A)**, TSS **(B)**, TA **(C)**, and ascorbic acid **(D)** of cherry tomato during storage at 25°C and 90% humidity. The asterisk symbolizes significant distinctions (*p* < 0.05) in comparison to the control. Triple replications in all experiments.

Figure 6 illustrates the effects of paeonol treatment on fruit weight loss, TSS, TA, and ascorbic acid in cherry tomato. Figure 6A showed that the weight loss of the control fruit and paeonol-treated fruit all increased with the extension of the storage period. The paeonol-treated fruit’s weight loss was significantly lower than the control group (*p* < 0.05) from the ninth to the fifteenth day.

TSS content of control fruit increased on the third day, then declined until the fifteenth day. TSS content of the paeonol-treated group increased until the sixth day, then decreased until the ninth day. A significant difference (*p* < 0.05) was observed between the paeonol treated-tomato and the control group from the sixth to the fifteenth day.

As displayed in Figure 6C, TA content decreased in control and paeonol treatments during the whole storage period, but no significant differences were discovered in the two treatments of cherry tomato.

The ascorbic acid content in the control group rapidly declined during the whole storage time, while the paeonol-treated group exhibited a minor decrease (Figure 6D). Further comparison found that the ascorbic acid content in paeonol-treated group was observably higher than those in the control group.

## 4 Discussion

As previously reported, paeonol could effectively inhibit the growth of bacterial cells, such as *Escherichia coli*, *Staphylococcus* spp., *Salmonella* spp., *Bacillus subtilis*, *Klebsiella pneumonia*, *Enterobacter cloacae*, *Listeria monocytogenes*, and others (Song, 2012; Zeng et al., 2022; Qian et al., 2021). However, reports of paeonol inhibiting fungal cell growth are limited to *Candida albicans* (Qian et al., 2022) and *Aspergillus flavus* (Li et al., 2022), and research on *B. cinerea* has not yet been reported. In the present study, we determined that paeonol inhibited mycelial growth and spore germination of *B. cinerea*, with a MIC value of 250 mg/L for paeonol against *B. cinerea*. The effect of paeonol on *B. cinerea* was further illuminated by SEM, which shows distortion and collapse of the fungal mycelia treated with paeonol (Figure 2). These results indicated that paeonol showed a potent inhibitory effect on the growth of *B. cinerea in vitro*. We also found that paeonol alleviated the severity of gray mold, caused by *B. cinerea*, in cherry tomato fruit. Compared with the results of paeonol treatment *in vitro*, the complete inhibitory concentration of paeonol *in vivo* (2,000 mg/L) was higher than that *in vitro* (250 mg/L). It may be explained by the fact that environmental conditions at the wound site are more complex and conducive to the growth of *B. cinerea* when compared to the PDB medium. This phenomenon was observed in luteolin, *ε*-polylysine, and hinokitiol studies by Liu et al. (2021), Jiao et al. (2020), and Wang et al. (2020), respectively. Paeonol’s complete inhibitory concentration against gray mold in fruit was lower than vanillin (Yang et al., 2021) or magnolol (Cui et al., 2021), suggesting that the former may be more suitable for controlling gray mold in agriculture.

Most antifungal agents target the cell wall and membrane of fungi (Pereira et al., 2015; Teixeira et al., 2023). Therefore, the mechanism of paeonol action was tested to determine whether paeonol antifungal activity involved direct interaction with the cell wall structure of *B. cinerea* (via sorbitol testing) and/or with the membrane structure of *B. cinerea* (via ergosterol testing) (Biernasiuk et al., 2021). Our results found that paeonol targets the cell membrane rather than the cell wall (Table 2). At the same time, we clearly showed that paeonol would destroy the integrity of cell membranes of *B. cinerea*, as leakage of cellular contents, including nucleic acids, proteins, potassium ions, and calcium ions, was significantly observed (Figure 4). A similar result was also confirmed by Li Q. et al. (2021), who discovered that paeonol strongly destroyed the integrity of cell membranes, leading to the leakage of cellular components in *A. flavus*. Ergosterol, pivotal in preserving cell functionality and integrity, is the main sterol component of the fungal cell membrane (Dupont et al., 2012). Previous studies have shown that plant-derived natural bioactive compounds, such as citral and perillaldehyde, can interfere with the ergosterol biosynthetic pathway or diminish ergosterol content (Cui et al., 2024; Meng et al., 2024). In our study, paeonol treatment reduced ergosterol content (Figure 4). Furthermore, RT-PCR results showed that ergosterol biosynthesis enzyme genes (*Erg1*, *Erg3*, and *Erg10*) were down-regulated in the presence of paeonol. The above results indicated that paeonol inhibited the expression of ergosterol biosynthesis enzyme gene, diminished ergosterol content, and disrupted cell membrane integrity of mycelia, leading to leakage of nucleic acids, proteins, potassium ions, and calcium ions.

In addition, several physicochemical parameters (e.g., water loss, TSS, TA, ascorbic acid content) were evaluated to establish the impact of paeonol on the quality of cherry tomato fruit, which is crucial to consumer satisfaction. In this research, paeonol exhibited no appreciable influence on the TA content of cherry tomato. Conversely, it can aid in maintaining the moisture, TSS, and ascorbic acid content. Water constitutes the major component of tomato fruit, maintaining cell turgor during growth and postharvest storage, along with fruit texture and appearance (Lai et al., 2018). TSS content can affect the taste of tomato fruits, which is a quality attribute very appreciated by consumers (Zahid et al., 2024). Ascorbic acid, a vital plant metabolite, functions as a cell-signaling modulator, antiradical agent, and enzyme cofactor in various critical physiological processes, including cell wall biosynthesis, secondary metabolites and phytohormones, stress resistance, photoprotection, cell division, and growth (Wolucka et al., 2005). The aforementioned findings indicated that the paeonol treatment could delay water loss in cherry tomato fruit, maintain higher contents of TSS and ascorbic acid, and culminate in retaining higher quality in the pulp of harvested.

## 5 Conclusion

In conclusion, paeonol could effectively inhibit the growth of *B. cinerea in vitro*. The possible mechanism was that paeonol targets the cell membrane, which downregulated ergosterol synthase genes, inhibited ergosterol synthesis, damaged cell membrane integrity, and led to cell death. Meanwhile, paeonol could control the gray mold caused by *B. cinerea* in cherry tomato fruit and prolong the shelf life of cherry tomato fruit. Overall, this potent action of paeonol on the management of gray mold and extension of the shelf life of postharvest fruit positions it as a promising candidate for advancement as a substitute for synthetic fungicides.

## Data Availability

The original contributions presented in the study are included in the article/supplementary material, further inquiries can be directed to the corresponding authors.
